# Comorbid Pathologies and Their Impact on Dementia with Lewy Bodies—Current View

**DOI:** 10.3390/ijms26167674

**Published:** 2025-08-08

**Authors:** Kurt A. Jellinger

**Affiliations:** Institute of Clinical Neurobiology, 1150 Vienna, Austria; kurt.jellinger@univie.ac.at

**Keywords:** dementia with Lewy bodies, Alzheimer pathology, concomitant pathologies, α-synuclein, biomarkers, neuroimaging

## Abstract

Dementia with Lewy bodies (DLB), the second common primary degenerative neurocognitive disorder after Alzheimer disease (AD), frequently presents concurrent co-pathologies that impact clinical presentation and progression. Neuropathological studies have demonstrated a high prevalence of coexistent AD-related neuropathological changes (ADNC), TAR DNA-binding protein 43 (TDP-43) proteinopathies, and cardiac and aging-related disorders, while frontotemporal lobar degeneration (FTLD) and tau-related syndromes play a minor role as DLB-related co-pathologies. Cerebrovascular lesions, including cerebral amyloid angiopathy, are the most prevalent non-neurodegenerative co-pathologies. Cardiovascular disorders, hypertension, and hyperlipidemia are also frequent comorbidities. Due to their high prevalence and clinical impact on DLB patients, clinical trials should account for these and other co-pathologies in their design and selection. Evaluation of these co-pathologies using and interpreting biomarkers may allow greater clinical diagnostic accuracy and the opportunity to better predict clinical progression. Therefore, there is an increasing need for biomarkers in dementia research. This review discusses the kind and frequency of the different co-pathologies in DLB and their clinical impact. It evaluates the possible value of disease-specific biomarkers and how they are helpful in the assessment and prevention of DLB and its co-pathologies.

## 1. Introduction

Dementia with Lewy bodies (DLB), the second most common neurodegenerative disorder in the elderly following Alzheimer disease (AD), is caused by abnormal α-synuclein (αSyn) forming intracytoplasmic Lewy bodies (LBs) and Lewy neurites in many nervous and extraneuronal systems. Clinically, this heterogeneous disease represents a wide spectrum of cognitive, sleep, motor, and autonomic symptoms. Besides cognitive impairment (CI) often preceding parkinsonism, DLB patients need to have at least two of the following four clinical features: visual hallucinations, fluctuating cognition, REM sleep behavior disorder (RBD), and parkinsonian symptoms, including rest tremor, bradykinesia, and postural instability [1]. DLB and Parkinson disease with dementia (PDD) are the two main Lewy body dementias that show many clinical, genetic, and morphological similarities but also essential differences [2,3,4,5]. According to international consensus, DLB is differentiated from PDD when CI precedes or concurrently occurs together with parkinsonism, while PDD is diagnosed when dementia begins at least one year after established motor parkinsonism [6,7]. This Consortium “one-year rule”, based on temporal differences between the onset of dementia and parkinsonism [1], has been confirmed by later neuropsychological studies [8]. Although it has been criticized as arbitrary, it is used as a pragmatic and effective tool for splitting the continuum between the two entities [9]. The spectrum of clinical syndromes with Lewy pathology (LP) and its phenotypes has been critically reviewed recently [10].

Like other neurodegenerative diseases, co-pathologies and comorbidities have been recognized in DLB that show different distribution patterns and injury mechanisms, further adding to the heterogeneity of brain changes [11]. They may be either coincidental or defined by the deposition and mutual interaction of specific misfolded proteins [12,13,14,15]. Co-pathologies have an impact on the disease development and/or its clinical presentation and progression by confounding brain dysfunction [11,16]. A majority of DLB patients have two or three concomitant disorders, and neuropathological studies demonstrated the high prevalence of concomitant Alzheimer-related neuropathological changes (ADNC), TAR DNA-binding protein of 43 kDA (TDP-43) [17,18], and other neurodegenerative conditions, including limbic predominant age-related TDP-43 encephalopathy (LATE) [19,20]. Cerebrovascular diseases are the most frequent non-neurodegenerative co-pathologies with multiple manifestations [21,22]. Total co-pathology prevalence varies across groups and was increased in neocortical DLB (70–81%), with amyloid-β (Aβ) (80%) and TDP-43 (22%) [14]. Further co-pathologies include argyrophilic grain disease and ageing-related tau astrogliopathy (ARTAG) [11], while coexisting Pick disease or motor neuron disease has not been observed [9]. Increasing age and APOE ε4 status are risk factors for co-pathologies independent of neurodegenerative disease. On the other hand, their severity may influence co-pathologies, as evidenced by their prevalence in neocortical DLB and intermediate ADNC due to interaction between αSyn strains and Aβ and tau proteins [23,24]. Assessment of clinical features and coexisting syndromes in DLB patients indicated a high comorbidity and geriatric syndrome burden [25]. The comorbidity rates of hypertension and hyperlipidemia in DLB patients were higher in females than in males [26]. The major co-pathologies in DLB are presented in Table 1.

This article is intended to review the current knowledge about co-pathologies and comorbidities in DLB and their cross-sectional and longitudinal clinical impact, as well as the possible application of disease-specific biomarkers and their assessment in DLB individuals. It will not include CI and behavioral disorders in DLB, which have been reviewed recently [4,27,28]. The literature research strategies are described in a Supplementary Materials.

**Table 1:** Co-pathologies in dementia with Lewy bodies (DLB).

Type of Disorder	Pathology	Prevalence and Specificity in DLB
Alzheimer disease	Amyloid and neuritic plaques, tau neurofibrillary tangles	28–89% of DLB patients have intermediate or high-level ADNC vs. 35–62% in PDD, and 10% in PDND [29,30,31,32]. ADNC is associated with higher cortical Lewy pathology burden [33].
TDP-43 pathology	TDP-43	13–60% of DLB patients have TDP-43 pathology vs. 19% PDD and 7.2% PDND [17,18,34,35]. It is more prevalent in advanced cortical Lewy pathology and DLB + AD [18]. In DLB, it follows distribution patterns of LATE [36].
LATE	Tau	Distribution pattern different from that in DLB and AD.
FTLD tau	4R tau	ARTAG 72% in DLB + AD cases [37]. AGD in few DLB cases [38]. CBS and PSP only rare.
Cerebrovascular pathology	Microbleeds	30–45% in DLB vs. 26.1% in PDD and 11.5% in PDND [39,40], associated with hypertension and CAA.
Cerebrovascular pathology	Cerebral infarcts	26.9% microinfarcts, 6.7% large infarcts (neuropathology) [41], 18.8–27% in MRI [42,43].
Cerebrovascular pathology	CAA	95% in DLB, 50% PDD, 24% PDND [5,44].
Cerebrovascular pathology	White matter hypertension	Due to hypertension, more frequent than in controls [43,45,46].

FTLD: frontotemporal lobar degeneration; ARTAG: ageing-related tau astrogliopathy; PSP: progressive supranuclear palsy; LATE: limbic-predominant age-related TDP-43 encephalopathy; PDND: Parkinson disease without dementia; AGD: argyrophilic grain disease; PDD: Parkinson disease with dementia; CAA: cerebral amyloid angiopathy.

## 2. DLB and Concomitant Alzheimer Disease Pathology

Alzheimer disease (AD) co-pathology (intermediate or high ADNC), defined by a higher burden of tau neurofibrillary tangles (NFTs) and Aβ or neuritic plaques, is present in 28–89% of DLB individuals, with a higher prevalence compared to both Parkinson disease (PD) (around 10%) and PDD (35%) [29,30,31,32,47,48]. It has been suggested that up to 50% of PDD patients and 28–89% of DLB cases have sufficient ADNC for a (second) neuropathological diagnosis of AD [49,50,51]. In particular, tau pathology has been linked to a greater LP burden [33,37,52] and was the only co-pathology considered when interpreting neuropathological findings in DLB that were recognized in the DLB guidelines [1,53]. Furthermore, total Aβ pathology scores correlated positively with total αSyn scores (*p*= 0.001). The factors that correlated best with the amount of αSyn pathology were the severity of Aβ load and the presence of the MAPT-H1 haplotype as contributing factors in DLB pathogenesis. LP modifies the risk factors for cerebral amyloid angiopathy (CAA) when present along with AD co-pathology [54]. Tau and αSyn frequently co-localize in limbic areas [55], and co-localization of phosphorylated tau (p-tau) and αSyn could be observed within astrocytes of AD cases with LBs [56]. Neocortical αSyn load was significantly higher in DLB cases with intermediate-to-high than in those with low-to-none ADNC co-pathology. Additional burden from the presence of other concomitant pathologies and synergistic effects of pathological proteins contributed to rapid disease progression [57]. Distinct αSyn patterns were apparent among DLB, mixed DLB/AD, and PDD that may relate to different spreading patterns in combined DLB and could explain some of the disease heterogeneity [58].

DLB/AD+ had more global and parietal gray matter atrophy compared to DLB/AD– cases [59]. They had decreased volume/thickness in all medial temporal lobe (MTL) subregions except posterior hippocampus; atrophy in multiple MTL subregions was associated with tau pathology load and memory performance. Smaller differences were seen in the parahippocampal cortex and anterior hippocampus. The observed pattern of atrophy largely follows expectations from NFT Braak stages [60]. MRI indicated hippocampal atrophy, and 18F-FDG PET showed hypometabolism in the MTL. Detection of Aβ pathology and tau load confirmed the concurrent presence of ADNC [61]. Although medial temporal atrophy can be supportive in distinguishing between DLB and AD [62,63], in DLB/AD+ cases, it is more severe than in DLB/AD − ones [59,64]. However, this has seldom been investigated together with biomarkers of AD or cerebrovascular co-pathologies [65]. Medial temporal atrophy in DLB depends on Aβ-related pathology [66]. Posterior basal forebrain atrophy is most severe in mixed DLB/AD compared to pure AD, whereas pure hippocampal atrophy is primarily associated with ADNC independent of LB pathology, with LB and AD pathology both contributing to cholinergic forebrain degeneration [67]. Moreover, DLB/AD+ patients had significantly lower 123I-FP-CIT SPECT bindings in the left amygdala and right hippocampus, indicating serotonergic deficits in limbic brain regions [68].

Potential confounders in the relations between DLB and AD pathologies include neuroinflammation and other changes, such as posterior hypometabolic mismatch related to atrophy, possibly reflecting impaired neuronal integrity caused by the impact of multiple proteinopathies [69]. Neuroinflammation is associated with AD co-pathology in DLB, with reactive and amoeboid microglia being common in mixed DLB/AD, while a higher density of swollen astrocytes was observed in pure AD cases but not in mixed DLB/AD or pure DLB cases. DLB/AD+ cases had higher CD68 loads in the amygdala and parahippocampal gyrus than pure DLB ones but did not differ in astrocytic loads. In these cases, microglial load associated strongly with Aβ and p-tau and only minimally with αSyn load. These findings provide novel insight into the molecular neuropathology of DLB, highlighting the importance of microglial activation in DLB/AD+ cases [70]. Furthermore, APOE ε4 and polygenic risk scores for AD are associated specifically with DLB/AD+ but less with DLB-pure, while the GBA p.E365K variant is associated with DLB-pure but less with DLB/AD+, indicating the heterogeneity in DLB [71].

The effects of amyloid and tau co-pathology in DLB (DLB/AD+) are associated with accelerated cognitive and functional decline, more frequent hallucinations, and higher mortality [72,73,74]. Patients with multiple etiology dementia, such as DLB/AD+, have faster disease progression, although their diagnosis can be challenging given the overlapping clinical and imaging features.

## 3. DLB and TDP-43 Pathology

Cytoplasmic inclusion bodies containing phosphorylated and truncated forms of TDP-43 are morphological hallmarks of amyotrophic lateral sclerosis (ALS) and a subset of frontotemporal lobe degeneration (FTLD-TDP), but they have also been identified in AD [75] and in other age-related neurodegenerative disorders, referred to as limbic-predominant age-related TDP-43 encephalopathy (LATE) [36]. TDP-43 pathology initially involves the amygdala, then progresses via limbic areas to the neocortex [76]. Accumulation of the TDP caspase cleavage product antibody (TDPccp) has been observed in LBs in PD and DLB, where it was co-localized with an antibody to αSyn, a general marker for LBs. TDPccp even detected a greater number of LBs than the αSyn antibody [77]. The synergistic association of αSyn and TDP-43 promotes the aggregation of both proteins and enhances neurodegeneration in vitro and in vivo [78,79], while αSyn aggregates have a synergistic effect on TDP-43 and tau aggregation [80]. Moreover, the accumulation of TDP-43 and mutant αSyn induces dopaminergic neurodegeneration, although the precise mechanisms are unclear [81].

TDP-43-positive inclusions within neurons and oligodendroglia were found not only in AD brains but also in DLB, as well as in FTLD with ubiquitin-positive inclusions, but not in cases of PD, progressive supranuclear palsy (PSP), corticobasal degeneration, Pick’s disease, or frontotemporal dementia and parkinsonism linked to chromosome 17 (FTDP-17). Immunohistochemical analysis of TDP-43 in pathologically confirmed cases demonstrated it in DLB/AD+ (31.3%), PD (7.2%), and PDD (19%), while DLB and normal controls exhibited none, and only one case presented TDP-43 pathology, respectively [34]. Others reported that 13–60% of DLB patients show TDP-43 pathology [17].

The amygdala and hippocampus that are vulnerable to tau and αSyn pathology are more severely affected by TDP-43 pathology in DLB and AD than in frontotemporal lobar degeneration with ubiquitin-positive but TDP-43-negative inclusions (FTLD-U). Thus, local distribution of TDP-43 pathology in DLB and AD was obviously different from that in FTLD-U cases; in DLB, it follows a distribution pattern consistent with that of LATE [36]. A subset of TDP-43-positive inclusions coexisted with both LBs and NFTs in the same neurons. While TDP-43 was rarely superimposed with tau, it was partly superimposed with αSyn, suggesting that neither NFTs nor LBs themselves showed TDP-43 immunoreactivity. However, the coexistence of TDP-43, tau, and αSyn pathologies may be related in some way to LB and AD pathology [17]. TDP-43 pathology burden is associated with greater LP burden and the presence of AD co-pathology, exemplifying the complex interrelation between the different neuropathologies [11,18].

## 4. DLB and Argyrophilic Grain Disease

Argyrophilic grain disease, an age-related 4R-tauopathy affecting the MTL that has been associated with psychiatric symptoms in PD [82,83], has been reported in two autopsy cases of DLB, the clinical course of which was consistent with DLB, including visual hallucinations, resting tremor, rigidity, and prominent psychiatric symptoms [38,84].

## 5. DLB and Other Neurodegenerative Disorders

FTLD-tau is rare and plays a minor role as a DLB co-pathology, whereas ARTAG is prevalent in DLB [37]. Coexistence of corticobasal syndrome (CBS) with pure DLB has been described once [85], while a rare form of DLB clinically manifesting as CBS was younger at disease onset and had less RBD than classical DLB. Among 523 autopsy-confirmed cases of DLB, three had concomitant PSP and four had concomitant AD as probable correlates of clinical features of CBS [86], as was seen in another CBS phenotype [87]. While reduction of nuclear density was noted in DLB neurons, astrocytes, and microglia, and in PSP astrocytes and microglia [88], DLB patients exhibit homogenous brainstem atrophy and can be distinguished from those with PSP by automated volume ratios [89].

## 6. DLB and Cerebrovascular Pathology

The effects of cerebrovascular lesions (CVLs) in DLB are not fully understood. Prevalence reports of cerebrovascular disease (CVD) in DLB are scarce, but autopsy studies reported a frequency of 20.2–34.4% [21,90]. CVLs are commonly assessed through white matter hyperintensities (WMHs) [91], which are associated with poorer cognition in DLB [65,92]. In DLB, 43% had WMH load, which was significantly higher than in PDD (30%), AD (27%), and normal controls (14%), but lower than that in vascular dementia (62%). In DLB, WMHs were associated with medial temporal atrophy, and this association was stronger than in cognitively unimpaired and AD [66]. In a series of 25 autopsy-proven DLB cases, nine (36%) were complicated by cerebral hemorrhages, while ten (40%) had microinfarcts, which were less common than in AD [93]. There are inconsistent findings regarding the prevalence of cerebral infarcts in DLB compared to controls [43]. In general, DLB patients had a higher risk of ischemic stroke compared to AD [94]. Cerebrovascular pathology inversely correlates with LP [41] and DLB clinical features [42], which indicates that less severe LP may be necessary for the development of CI in the presence of cerebrovascular co-pathology. CAA shows a high prevalence in DLB, being more common than in PDD and non-demented PD (PD-ND) (95% vs. 50% and 24%) [4,5,44]. Similarly, cerebral microbleeds (CMBs) are more frequent in DLB, followed by PDD, DLB with mild CI, and PD-ND [40,95,96]. A recent systematic review and meta-analysis found that the prevalence of CMBs across AD, DLB, and PDD was highly influenced by the MRI techniques used. They may be associated with hypertension (100%) compared to those without (70%) and with hypertensive small vessel disease [97], while they may not be associated with Aβ deposition [98]. CMBs in AD are associated with a history of hypertension and Aβ burden. In contrast, indicating different underlying pathophysiology [99], CMB prevalence in DLB was highly variable but broadly similar to AD (0–48%), with a lobar predominance. CMB frequency in DLB (30–45%) was high vs. 26.1% in PDD, 11.5% in PD-ND, and 17% in controls [39,40]. No relationship was found between large cortical or small subcortical infarcts or intracerebral hemorrhages in DLB [22].

A recent review showed an increased severity of WMHs on MRI, but not neuropathology, in DLB compared to PD-ND. WMHs were larger in AD than in DLB. The prevalence of hypertension ranged from 21% to 56% in DLB and from 30% to 52% in AD, while some research suggested that DLB patients have greater rates of diabetes mellitus (DM) (18.7–37%) than AD ones (9–17.5%). Thus, WMH volumes can differentiate between these diseases [100]. On the other hand, DLB patients show CVD-related disruptions of cholinergic white matter (WM) (external capsule and cingulum) that may be linked to frontal atrophy [101].

A current discussion about the pathogenesis of WMH is whether they are considered a marker of cerebrovascular disease or other etiologies, including axonal loss and retrograde degeneration due to AD-related cortical pathology [102,103].

While previous studies demonstrated an association between enlarged perivascular spaces and AD, the association with DLB was only recently clarified. In multivariable logistic regression analyses, enlarged perivascular spaces in the basal ganglia were independently associated with DLB. High pulse wave velocity was also independently associated with these lesions in both the basal ganglia and centrum semiovale, leading to accelerated formation in DLB [104].

## 7. Cardiovascular Disease and Cardiovascular Risk Factors

DLB is associated with an increased risk of atrial fibrillation (22.3%) [105]; sick sinus syndrome (SSS) and high-degree atrioventricular block are more common in DLB than in AD (2.2% vs 1.5%; *p* = 0.008), as is pacemaker implantation secondary to SSS [106]. Carotid sinus syndrome, a common cause of syncopes in elderly persons, is frequent in DLB, causing hypotension and deep WM lesions due to microvascular pathology [107]. Moreover, DLB patients show a high prevalence of atrial conduction abnormalities: 58% exhibited pathological P wave terminal forces, 12% in the AD group, and 81% exhibited pathological P wave duration vs. 52% in the AD group. The clinical significance of pathological P wave parameters in DLB is unknown, but their presence suggests an atrial cardiomyopathy that could be due to αSyn deposition, autonomic dysfunction, or a combination thereof. This indicates cardiac complications, in particular, disease-related atrial conduction disorders, in DLB [108]. DLB further increases the risk of orthostatic hypotension by 3.62- to 7.71-fold compared to healthy controls [109], with postprandial hypotension being the most common finding of cardiovascular autonomic dysfunction [110]. While DM and, to a lesser extent, hypertension influence survival in AD, this has not been observed in DLB, which, independently of the presence of vascular risk factors, seems to be a more aggressive disorder than AD when survival time and mortality are taken into account [111]. A low prevalence of cardiovascular disease and risk factors, including DM and hypertension, argues against their contribution to the development of this neurodegenerative disease [112]. Among vascular risk factors, DM was associated with a lower increase in dementia rating scale scores in DLB [113].

US studies, by combining epidemiological and genomic data, demonstrated significantly reduced DLB risk associated with drugs used to treat cardiovascular diseases (anti-hypertensives, anti-diabetics, cholesterol-lowering agents) that were associated with a larger risk reduction among Black DLB patients compared to other racial groups [114]. Recent studies about the causal relationship between lipid metabolism and DLB revealed that elevated low-density lipoprotein cholesterol (LDL-C) and remnant cholesterol are significant risk factors for DLB [115].

Glycerophospholipids play a crucial role in the pathogenesis of DLB, but the specific components that play a role differ from those with the APOE ε4 carriers [116]. On the other hand, the results of a bidirectional two-sample Mendelian randomization study did not support the hypothesis that fatty acids could reduce the risk of developing DLB, since they showed that no fatty acids were associated with the incidence of DLB [117]. These results have potential implications for informing dietary recommendations and treatment.

## 8. Other Comorbidities

In comparison to the high frequency of multiple comorbidities in PD [28], studies of the available literature revealed only a few coincident DLB and other disorders, e.g., rare comorbidity with autoimmune disorders like myasthenia gravis [118] or Hashimoto encephalopathy [119], whereas there is a significant overlap between anti-IgLON5 disease, an autoimmune encephalitis, and DLB [120]. While the association between Down’s syndrome and AD is well established, its combination with DLB appears to be rare [121]. Comorbid idiopathic normal pressure hydrocephalus was reported in 10.1% of DLB patients [122]. Older patients with DLB may develop anemia, which is associated with the presence of congestive heart failure [123].

Finally, patients with bipolar disorder have been found to have an elevated risk of developing PD and/or DLB, which may be attributed to dopamine dysregulation resulting from multiple relapses [124]. Several reports have suggested that attention-deficit/hyperactivity disorder (ADHD) may be a risk factor for DLB, which has been confirmed, showing that adult ADHD is independently associated with an increased risk of DLB [125].

## 9. Clinical Impact of Co-Pathologies in DLB

Concomitant pathologies and comorbidities are not only prevalent in DLB but may influence the clinical presentation and disease progression by compromising brain function. Therefore, their individual clinical impact should be proven and evaluated to predict manifestation, complications, and clinical progression in order to understand outcomes and effects of treatment. AD is the co-pathology with the greatest clinical impact, and Aβ and tau have been related to cognitive decline [126,127,128,129,130]. The presence of AD co-pathology in individuals with DLB associates with differences in clinical manifestation and progression [73,131]. As the degree of ADNC increases, patients with LP are increasingly likely to have a DLB clinical phenotype (early dementia compared to PDD) [132]. AD co-pathology also affects the frequency of core symptoms, with less frequent parkinsonism and RBD in those with DLB and higher Braak NFT stages [126,133]. DLB/AD+ patients show worse global cognition, especially in attentive/executive and visuospatial functions, and worse motor functions than those with no evidence of AD [134,135,136]. Hallucinations are more common in DLB/AD+ in one study [72], but not in others [126,133]. Memory performance is worse in DLB/AD+ patients compared to DLB/AD − ones, also after correction for age and sex. Hallucinations are more frequent in DLB/AD+ [72]. DLB and ADNC showed worse antemortem cumulative cognitive deficits, while cortical DLB exhibited greater executive/visuospatial deficits than the ADNC-only group [137]. The age of onset of dementia is lower in DLB patients with higher tau and Aβ load [138]. DLB patients with MTL atrophy had abnormal cerebrospinal fluid (CSF) Aβ42, shorter disease duration, and older age. Those with posterior atrophy had abnormal levels of CSF Aβ42 and p-tau, older age, lower education, and shorter disease duration. Global cortical atrophy was associated with male sex, lower education, and older age, but not with any AD-related CSF biomarkers. This indicates a potential combined effect of Aβ and tau-related pathologies on the integrity of posterior cortices in DLB brains, whereas only Aβ seems to be elevated in cases with medial temporal atrophy [66,139].

Concurrent ADNC, and particularly tau accumulation, significantly impact both cognitive function and glucose metabolism in DLB, which underscores the importance of addressing AD-related changes in its clinical diagnosis and management [140]. DLB/AD+ patients have a higher nursing home admittance risk and a higher mortality than DLB/AD − ones [72]. Shorter survival appears to be linked with increasing Aβ load [141,142], with a lower impact of tau pathology [141]. Furthermore, the severity and distribution of LP appear important, since diffuse neocortical and occipital LP showed a more rapid disease course than those involving the brainstem and limbic areas [33,141].

Limited data exist on the implications of TDP-43 pathology on clinical phenotype in DLB. Although its presence is also associated with older age and a higher likelihood of tau deposition, a lower likelihood of DLB diagnosis in individuals with Lewy and TDP-43 pathology occurred, even when considering these concomitant factors. Autopsy-confirmed DLB cases with TDP-43 co-pathology furthermore had a lower likelihood of presenting visual hallucinations and parkinsonism and were, therefore, less frequently diagnosed during life [143].

As mentioned above, CVLs in DLB correlate negatively with the severity of LP [41,144]. This association is consistent with CVLs lowering the threshold for dementia in patients with Lewy and AD pathologies [21], whereas the evaluation of associated WMHs has led to conflicting results. Some studies emphasized the importance of WMH, which affects cholinergic WM pathways [42,101,145], or showed an inconsistent association of the WMH burden with visual hallucinations [42,146], while others found no association of WMH and cognition [45,147,148,149]. Parkinsonism and cognitive fluctuations were not associated with WMH burden [42].

## 10. DLB Comorbidities and Genetic Findings

DLB shares genetic findings with PD (αSyn /SNCA/ and β-glucocerebrosidase /GBA) [150,151] and AD (APOE) [150,151,152,153]. Different regions of SNCA have been associated with PD and DLB [150,154], while dysregulation of TMEM175 may confer PD and DLB risk and may be partly responsible for their comorbidity, indicating a common risk factor between both diseases [155]. APOE is associated with DLB associated with no or low ADNC burden [153], while it is associated with faster disease progression and shorter survival in DLB [156,157]. APOE ε4/TOMM-40 long poly-T repeat allele variants increase the susceptibility and risk of earlier DLB onset, an effect explained by concomitant ADNC, while it is not significant in DLB without AD [158]. Furthermore, a molecular interaction of PSEN1 and αSyn may explain the clinical and pathophysiological overlap between synucleinopathies, including DLB and some forms of AD [159]. Recent genome-wide association studies (GWASs) revealed a number of genetic associations, giving insight into the co-pathology of DLB and AD [160].

Higher risk for ADNC co-pathology is based on the age at disease onset and genotype on three single-nucleotide polymorphisms (SNPs) (APOE, BIN1, and SORL1 loci). Individuals with risk scores for ADNC have a four-fold increased likelihood of concomitant ADNC at autopsy compared with those in the lowest two quintiles. Thus, in patients with autopsy-confirmed DLB, three AD-risk SNPs and age at disease onset indicate concomitant ADNC, with implications for identifying DLB patients with Aβ and tau pathologies [161]. Other studies found that APOE ε4 and polygenic risk scores for AD are associated specifically with DLB/AD+ but less with DLB-pure. In addition, the GBA p.E365K variant showed a strong association with DLB-pure and less with DLB/AD+. APOE ε4 has been shown to be associated with reduced MMSE (mini-mental state examination) scores, higher odds being associated with fluctuations and lower disease duration. AD co-pathology is less prevalent in DLB individuals with GBA mutation [132]. In addition, the GBA p.E365K variant reduced the age at disease onset, while other variants and the polygenic risk score for AD did not associate with clinical features [71].

## 11. Contribution of Biomarkers for DLB Co-Pathologies

Biomarkers are proving to be useful predictors of DLB co-pathologies. They are essential both to support the accurate diagnosis of DLB and possible co-pathologies, to delineate the influence of coexisting pathologies, and to create subgroups most likely to derive benefits from disease-modifying therapies [74]. Biomarkers can be classified based on their modality into biofluid-, neuroimaging-, neurophysiology-, and tissue-based (biopsy) measures. Biomarkers can also be divided into disease-specific and non-specific ones according to the measured pathological feature. Disease-specific biomarkers include those that quantify specific changes of the underlying pathology, e.g., Aβ, tau, αSyn, and TDP-43. They have the potential to document the presence of specific co-pathologies, while disease-non-specific biomarkers measure changes that are not specific to a pathology, like brain atrophy, neuronal dysfunction, synaptic loss, or glial activation [11].

Despite the urgent clinical need, there is currently no reliable protein biomarker for DLB. A recent meta-analysis confirmed significantly lower CSF tau levels in DLB when compared to AD. Among 305 differentially abundant proteins (DAP), 16 were replicated in DLB, and six were confirmed (TAU, SYUA, NFL, CHI3L1, GFAP, and CLAT). These may contribute to DLB pathology by impacting misfolded protein clearance, dopamine neurotransmission, apoptosis, neuroinflammation, synaptic plasticity, and extracellular vesicles. A multiplex proteomic analysis using the recently released nucleic acid linked immunosandwich assay (NULISA™) platform revealed unique targets of DLB pathology and progression [162]. This indicates promising diagnostic biomarkers for DLB and may help prioritize molecular pathways for therapeutic target discovery [163].

A recent comparative proteomic analysis of postmortem brains offered valuable insights into the network-based biomarker characterization of a molecular signature of DLB, PDD, and AD. Reduced levels of the autophagy protein p62 (SQSTM1) differentiated DLB from AD, while DLB was distinguished from both PDD and AD by altered TRIM33 and cysteine/glutamate transporter (SLC7A11) levels across brain regions. Extracellular matrix proteins and members of the complement system (decorin, biglycan, C4A, C4B) showed a strong positive correlation with cognitive decline [164].

## 12. Biomarkers Indicating Concomitant AD Pathology

Concomitant AD pathology in DLB can be predicted in vivo by means of CSF, plasma, MRI, and PET biomarkers, whereas the most prominent technique to date for identifying LP is the αSyn seeding amplification assay (SAA) [165]. Pathological imaging (PET scan) and CSF AD biomarkers are associated with a higher likelihood of cognitive decline in DLB but do not always mirror the neuropathological severity as in pure AD. Implementing the use of blood-based AD biomarkers may allow faster screening of DLB patients for AD co-pathology, thus improving the diagnostic sensitivity for DLB/AD+ [166,167].

In individuals with DLB, there are already sufficient data indicating that AD biomarker results are valid and interpretable (Table 2). There is increasing clinical availability of AD biomarkers, which may be used in DLB patients during routine clinical examination. Positive AD biomarker results may align with the fact that approximately 20% of patients meeting previous versions of DLB diagnostic criteria [1] had no evidence of DLB, AD being the most common pathology of these cases, while 16% of AD patients with no evidence of synuclein pathology met criteria of probable DLB [133]. Given the high frequency of AD co-pathology in patients with DLB, it is likely that αSyn biomarkers would also be required to identify cases with DLB/AD+ other than DLB as the primary pathology [168]. In the prodromal stage, contrary to AD patients, in those with DLB, CSF biomarkers are not altered. At the demented stage of DLB, Aβ42 and Aβ40 levels are reduced, and the Aβ42/40 ratio remains unchanged between the prodromal and demented stages, contrary to what was observed in AD. Tau and p-tau 181 levels remained unaltered in prodromal stages of DLB [169]. CSF αSyn assays show a significant difference between DLB and AD, with αSyn levels being significantly higher in AD patients, which occurs already in the prodromal stage [170].

### 12.1. Cerebrospinal Fluid Biomarkers

Earlier correlation studies of CSF tau and Aβ levels in neuropathologically verified DLB with concomitant AD pathology showed that both the extent of NFT pathology (Braak stage) and AD stage were inversely correlated with CSF Aβ-42 levels, whereas CERAD stage (neuritic plaques) showed no significant correlation. CAA correlated positively with total tau and total tau (t-tau)/Aβ42 ratio and inversely with Aβ levels, but there was a heterogeneous extent of CAA in this group [171]. A substantial proportion of DLB patients had abnormal values for CSF Aβ42, t-tau, and p-tau, and a CSF AD profile was observed in 25% of DLB patients; these were older, performed worse on MMSE, and had a shorter disease duration compared with those with normal CSF [172]. Others reported that about 40% of DLB patients have the typical CSF AD biomarker profile [173]. A higher burden of abnormal CSF p-tau levels was reported to decrease the odds of presenting core DLB features [126,174,175]. An AD CSF profile and PET amyloid + tau burden have been associated with cognitive decline in DLB [176,177,178,179]. When examining biomarkers of co-pathology, amyloidosis was detected in 18%, 48%, and 71% and AD biomarkers in 0%, 8.7%, and 42.9% of DLB with different stages of CI, respectively. Co-occurring biomarkers for αSyn SAAs [180] and amyloidosis were present in 12% and 14% of AD compared to 43% and 57% of DLB patients with different stages of CI. These data show that using a combination of αSyn SAA and AD biomarkers can identify individuals with αSyn, ADNC, and co-pathology better than traditional clinical criteria alone [134,181].

Another study reported that CSF levels of homovanillic acid (HVA) and 5-hydroxyindoleacetic acid (5-HIAA) decreased with LP and were especially low in cases of DLB/AD+. The combination of HVA, 5-HIAA, and the proteins t-tau and Aβ-42 in CSF was useful for discriminating between DLB, DLB/AD+, and AD with high accuracy [182]. αSyn SAAs not only provide a specific marker for DLB but also allow the in vivo identification of co-occurring LP in AD, which is associated with CI and worse visuospatial and motor functions [134]. CSF-derived biomarkers of Aβ42 and t-tau/Aβ42 are also associated with neuropsychiatric symptoms in DLB/AD+ cases [29]. CSF VAMP-2 and SNAP-25 (attachment receptor proteins) are both decreased in pure DLB but increased in DLB/AD+ and showed good accuracy to discriminate both. Both proteins are associated with CSF p-tau and t-tau and with the Aβ42/40 ratio in DLB/AD+. SNAP-25 was associated with CSF neurofilament light chain (NfL) and with MMSE scores in DLB/AD+. Thus, CSF VAMP-2 and SNAP-25 are promising surrogate markers of synapse degeneration in DLB/AD+ [183].

### 12.2. Plasma Biomarkers

The newly developed plasma Aβ and tau assays will offer less invasive, easily deployable biomarkers [184,185,186]. Plasma p-tau-181 is closely related with ADNC, reflecting both tau and early Aβ deposition; it is discriminatory in pathologically confirmed AD, regardless of clinical presentation [187,188,189,190]. Plasma αSyn not only separates PD from DLB but may impact DLB clinical presentation, particularly cognition [191]. Plasma tau levels are elevated in DLB patients with pathological CSF Aβ values and faster cognitive decline [186]. Plasma p-tau-181 correlated with abnormal tau and Aβ PET and may identify co-morbid ADNC in DLB [192,193,194]. Plasma p-tau-181 correlates with AD CSF biomarkers, PET tau, and Aβ imaging, and its levels are higher than in controls [177,186,194,195]. This suggests that plasma p-tau-181 can be used as a marker of AD co-pathology in DLB. Pathological CSF Aβ and elevated plasma p-tau-181 and -217 are associated with cognitive decline, reflecting ADNC contribution [186,196]. No difference was found between DLB patients with and without a positive Aβ PET scan [193].

Plasma p-tau-181 and Aβ42/40 ratio predict conversion to DLB in idiopathic RBD [197]. Plasma p-tau-181 reflects tau and amyloid pathology in DLB and is a useful indicator for neurodegeneration in cortical regions vulnerable to NFT pathology, adding value to identifying AD co-pathology. This can be detected with plasma p-tau-181 and glial fibrillary acidic protein (GFAP) [198].

Plasma GFAP may be sensitive to concomitant ADNC in DLB, especially the accumulation of Aβ plaques [193]. Elevated GFAP in DLB shows preferential increases in postmortem and CSF Aβ+ individuals and has been associated with lower MMSE scores [193,196]. A combination of multiple plasma biomarkers (Aβ40/42, GFAP, NfL, and p-tau-181) has been shown to improve the accuracy of identifying DLB/AD+ [135,177].

### 12.3. Imaging Markers

Multiple structural and functional imaging methods have been evaluated in DLB [199] and have been used to evaluate differences in brain atrophy patterns in individuals with DLB with and without AD co-pathology [139,200]. DLB/AD+ had decreased volume/thickness compared to DLB/AD− in all medial temporal regions except posterior hippocampus. Atrophy in these regions is associated with memory performance and tau load. These observed patterns of atrophy largely follow expectations from AD Braak stages, except for posterior hippocampus. In the subset of patients with autopsy-proven lower volume of the entorhinal cortex, this was associated with higher tau load. Secondary analyses correlated MTL volume with cognition and neuropathology. Longitudinal studies are needed to validate the spread of neurodegeneration [60]. Amyloid PET was positive in 59–64% of individuals with DLB [73,201,202]. In a large multicenter DLB cohort using both amyloid PET and CSF, 15% of DLB patients were A+T+ (A—amyloid, T—tau), 32% were A+T−, and 13% were A−T+ [126,131]. Multimodal PET studies indicated that ADNC, and particularly tau accumulation, significantly impact both cognitive dysfunction and glucose metabolism in DLB, which underscores the importance of AD-related co-pathologies in the clinical course and management of DLB patients [140]. In a series of autopsy-proven cases of DLB (and PD), the best-fit generated risk score for ADNC based on age of disease onset and genotype at three SNPs (single-nucleotide polymorphisms) (APOE, BIN1, and SORL1 loci) provided a simple model incorporating three AD-risk SNPs for detecting concomitant AD pathology [161].

Tau-PET imaging in DLB shows cortical aggregates of tau, even in those without elevated Aβ levels. Tau deposition is associated with CI [203]. Medial temporal AV-1451 tau uptake distinguishes AD from DLB, in which elevated posterior temporoparietal and occipital AV-1451 uptake is associated with global PiB uptake, suggesting atypical patterns of tau deposition in DLB [178]. In DLB/AD+, MRI shows hippocampal atrophy and 18F-FDG PET hypometabolism in the MTL [61], while 18F-florzolotau PET imaging elucidates tau pathology patterns in DLB, with 54% showing patterns similar to AD, whereas 16% exhibit distinct patterns [204]. Structural changes in multiple regions and focal orientation dispersion index (ODI) reductions in the occipital cortex were seen in DLB with AD co-pathologies, where APOE genotype influenced Aβ levels and elevated tau, leading to microstructural injury. These findings highlight the role of ADNC in DLB neurodegeneration, advocating multi-target therapeutic approaches [205].

In conclusion, the utilization of AD biomarker assays in individuals with DLB has increased in research and clinical settings and has provided further insight into the importance of AD co-pathology in this disorder.

## 13. Non-Specific Biomarkers for DLB Co-Pathologies

Besides LB and AD pathology, biofluid and neuroimaging markers related to other disease mechanisms, such as CVD, neurodegeneration, synaptic loss, and neuroinflammation, have been used in DLB [22,206,207,208,209]. Furthermore, biomarkers for TDP-43 have been developed, but CSF assays have not yet been validated [210]. NfL is a disease-non-specific biomarker of axonal degeneration, which is elevated in plasma and CSF in early prodromal stages of DLB and associated with cognitive decline [211,212]. Other promising biomarkers of synapse dysfunction in DLB are those related to neurotransmitter transport and synapse secretion, such as VGF, PDYN, SCG2, and neuronal pentraxins (NPTX), which enhance diagnostic accuracy when combined with other biomarkers [208,213]. Non-specific biomarkers can be confounded by co-morbid neuropathology and may be useful in combination with other markers in diagnosis and prognosis [74]. A multilabel non-ADNC classifier in an autopsy-confirmed cohort using demographic, genetic, clinical, MRI, and ADNC variables (Aβ and tau) showed accuracies of 84% for TDP-43, 81% for DLB, and 81% to 93% for CAA. These results lead to a better understanding of the factors that lead to cognitive decline due to non-ADNC co-pathologies [214].

A review of disease-specific and non-specific biomarkers in the setting of DLB co-pathologies is given in Table 3.

## 14. Conclusions and Outlook

Concurrent co-pathologies have been repeatedly reported to be common in the great majority of individuals with DLB, as well as in other neurodegenerative disorders [11,16,215]. Most of them are more common than in normal elderly people and impact evolution, clinical presentation, progression, and outcome of the basic disorder. Some of the co-pathologies are present even before the manifestation of the typical signs and symptoms of DLB. However, our understanding of co-pathologies and comorbid disorders is limited because current evidence is mainly derived from retrospective autopsy studies. These demonstrated a high prevalence of coexistent ADNC and TDP-43 pathologies, including argyrophilic grain disease and ARTAG, which are frequent phenomena in old-aged subjects, while other coexisting neurodegenerative diseases, such as PSP and corticobasal degeneration, are extremely rare.

A high prevalence of CVLs, in particular microbleeds, CAA, microinfarcts, and WMHs in DLB, is related to concomitant hypertension, DM, and hyperlipidemia, which are more frequent than in the general population. For some of these age-related comorbidities, it is difficult to disentangle their individual contribution to disease progression, cognitive decline, or other clinical symptoms, but, in general, a combination of various co-pathologies is responsible for the progression of the basic disease and its complications [11,216,217].

**Figure 1:**
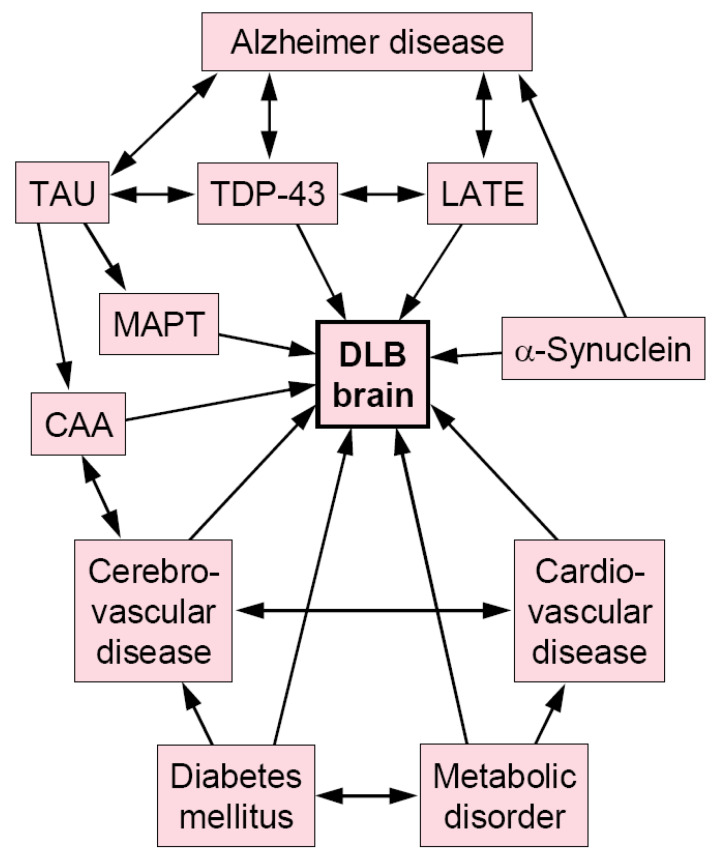
Hypothetical relationships between DLB and other pathologies. Arrows indicate causal influences between pathologies. DLB: dementia with Lewy bodies; LATE: limbic-predominant age-related TDP-43 encephalopathy; CAA: cerebral amyloid angiopathy; TDP-43: transactive response DNA binding protein of 43 kDa.

The frequent concurrence of various pathologies in DLB, in particular with ADNC and TDP-43 pathology, is probably due to a complex interaction of various pathological proteins, like tau, Aβ, αSyn, or TDP-43, that concur with aging [17,24,50,144,218] and affect multiple molecular pathways and interrelated pathobiological mechanisms. They result in a complex interplay of disease development, clinical symptoms, and disease progression in DLB (see Figure 1). Some of these complex mechanisms exert influence via genetic risk factors that involve protein cleavage and transport mechanisms, autophagy, and endosomal, lysosomal, mitochondrial, and other cellular functions [219], while the influence of environmental factors, lifestyle, and other exogenous influences is poorly known. However, cardiovascular and cerebrovascular risk factors, such as DM, hypertension, and hyperlipidemia, have an impact on the pathogenesis of many co-pathologies.

Apart from postmortem verification, major approaches to the early identification of co-pathologies are disease-specific biomarkers, the development of which is an essential approach for their early identification and evaluation of specific treatment modalities. It is now well established that the clinical presentation historically applied to define probable comorbidities was often not reflective of the underlying neuropathology. The same diagnostic difficulties are apparent in DLB, with comorbid pathologies contributing further to reducing diagnostic accuracy [143,220]. A biological definition is needed to define the contribution of co-pathologies. For instance, it is important to consider that strategies specific to DLB are needed to detect concomitant ADNC [221]. In vivo detection of AD co-pathology can give valuable prognostic information and may be useful in the design of clinical trials. Increasing evidence suggests that a combination of plasma biomarkers will have a high accuracy in detecting AD co-pathology in DLB [177]. Before these blood-based biomarkers can be incorporated into clinical practice, further validation is needed with standardizing assays and establishing appropriate cut-offs to ensure their reliability [186]. Future approaches will require integrated multi-modal biomarkers that characterize the prevalence, origin, impact, and progression of co-pathologies in prodromal and early disease stages. With αSyn testing available through CSF, plasma biomarkers, and skin biopsies, researchers will need to decide how best to share multiple biomarker results and their implications with individuals whose clinical presentations suggest DLB in order to exclude co-pathologies.

In addition to disease-specific biomarkers, like CSF, plasma, and PET biomarkers, as well as tissue biopsies to assess specific pathological proteins (tau, Aβ, αSyn, etc.), disease-non-specific biomarkers (neuroimaging, MRI, SPECT, electrophysiology, blood biomarkers like NfL, GFAP, etc.) are intended to reflect pathological changes caused by co-pathologies, like brain atrophy, neuronal and synapse dysfunction and loss, metabolic disorders, or biochemical abnormalities. These biomarkers are also of specific interest for the development and monitoring of disease-modifying treatments. Biomarkers will be critical in supporting early diagnosis and identifying patient-specific neuropathological footprints to facilitate a shift towards precision medicine with stratification of patients most likely to benefit from specific clinical trials. The proposed multi-modal biomarker approach will be important for the selection, evaluation, and interpretation of clinical results of prohibitional and therapeutic approaches to DLB and its co-pathologies, for which no cure is currently available.

## Figures and Tables

**Table 2:** Indications for using AD biomarkers in DLB (modified from [168]).

To test whether there is AD co-morbidity in individuals with DLB.
The planned test looks for AD-related changes in the brains of DLB patients.
Based on the fact that the clinical diagnosis of DLB can be wrong in about 20%, further diagnostic measures are necessary.
The planned test is necessary because it is known that more than 50% of DLB patients may have additional AD-related pathologies.
It should be considered that other brain changes can be present in the brain of individuals with DLB, such as cerebrovascular lesions and other brain diseases that should be excluded.
Comparison of clinical findings with AD and other biomarkers is necessary.
Comparison of the results of biomarker studies is necessary to clarify the clinical diagnosis and the implications for further treatment.
Information for patients and caregivers about the potential benefits of AD (and other) biomarkers for clarifying the clinical diagnosis is advisable.
Discussion with patients and caregivers about diagnostic, possible prognostic, and treatment implications of positive AD biomarker tests is advisable.
Information for patients and caregivers about the essentials and potential risks of AD biomarkers in the context of DLB is necessary, since suicide risk is more common in DLB than in AD dementia [165,166,167].

AD: Alzheimer disease; DLB: dementia with Lewy bodies. Table used under the terms of the http://creativecommons.org/licenses/by-nc/4.0/ License, accessed on 31 July 2025.

**Table 3:** Biomarkers in the setting of DLB co-pathologies (modified from [11]).

Pathological Change	Biomarker Modality	Implications in Dementia with Lewy Bodies
Disease-specific biomarkers		
Aβ	CSF/plasma Aβ40/42 PET Aβ	All modalities predict Aβ plaques in the brain and precede PET abnormalities.
Tau	CSF/plasma p-tau PET p-tau	Correlate with tau brain deposition.
αSyn	CSF/plasma αSyn (SAA) Skin, olfactory mucosa, submandibular gland, colon: Biopsy	Detection of the presence/absence of αSyn.
Limbic TDP-43 co-pathology	RT-QuIC	To detect in DLB.
Disease-non-specific biomarkers		
Brain atrophy	MRI	Correlates with the degree of cognitive impairment.
Neuronal dysfunction and damage Glial activation	CSF and blood biomarkers	Structural and functional imaging alterations strongly correlate with fluid-based biomarkers.
Synaptic loss	CSF and blood biomarkers	Fluid-based biomarkers are correlated with synaptic changes.
Structural and functional imaging biomarkers		
Relative preservation of medial temporal lobe structures	MRI CT	Included in the diagnostic criteria for DLB.
Reduced basal ganglia dopamine transporter uptake	SPECT	
Reduced occipital metabolism	FDG-PET	
Medial temporal lobe structures atrophy	MRI	AD co-pathology (and TDP-43) are associated with greater medial temporal lobe atrophy in DLB.
Abnormalities in the cholinergic system	MRI	Atrophy of nucleus basalis of Meynert.
Limbic TDP-43 co-pathology	RT-QuIC	Studies are needed in DLB.
Higher WMH burden	MRI	Associated with more neurodegeneration in DLB.
Structural connectivity changes	Diffusion tensor imaging (DTI)	Studies are needed in DLB.
Temporoparietal and occipital hypometabolism	Fluorine-18-fluorodeoxyglucose (FDG) PET	Characteristic of DLB.
Abnormalities in serotonergic systems	Volumetric measures and PET radiotracers	Further studies are needed in DLB with co-pathologies.
Neuro-axonal damage	Neurofilament Light (NfL)	Elevated already in prodromal DLB stages, higher levels in the presence of AD co-pathology.
Glial-related change	Glial fibrillary acidic protein (GFAP), the soluble triggering receptor expressed on myeloid cells 2 (sTREM2-microglia).	Elevated in DLB compared to controls.
Synaptic dysfunction	Fluid-based biomarkers: synaptosomal-associated protein 25 (SNAP-25), Synaptogamin-1, and neurogranin.	Further studies are needed in DLB with co-pathologies.

DLB: dementia with Lewy bodies; αSyn: α-synuclein; SAA: seeding amplification assay; CSF: cerebrospinal fluid; RT-QuIC: real-time quaking-induced conversation.

## Data Availability

No new data were created or analyzed in this study. Data sharing is not applicable to this article.

## Supplementary Materials

The following supporting information can be downloaded at: https://www.mdpi.com/article/10.3390/ijms26167674/s1. Reference [222] is cited in the Supplementary Materials.

## Abbreviations

The following abbreviations are used in this manuscript:

αSyn	α-Synuclein
AD	Alzheimer disease
ADNC	Alzheimer-related neuropathological changes
ARTAG	Ageing-related tau astrogliopathy
Aβ	Amyloid-β
CAA	Cerebral amyloid angiopathy
CBS	Corticobasal syndrome
CI	Cognitive impairment
CMBs	Cerebral microbleeds
CSF	Cerebrospinal fluid
CVD	Cerebrovascular disease
CVLs	Cerebrovascular lesions
DLB	Dementia with Lewy bodies
DM	Diabetes mellitus
FTLD	Frontotemporal lobe degeneration
GFAP	Glial fibrillary acidic protein
LATE	Limbic predominant age-related TDP-43 encephalopathy
LB	Lewy body
LP	Lewy pathology
MTL	Medial temporal lobe
NfL	Neurofilament light chain
NFTs	Neurofibrillary tangles
PD	Parkinson disease
PDD	Parkinson disease with dementia
PD-ND	Non-demented PD
PSP	Progressive supranucleal palsy
p-tau	Phosphorylated tau
RBD	REM sleep behavior disorder
SAA	Seeding amplification assays
SNPs	Single-nucleotide polymorphisms
TDP-43	TAR DNA-binding protein of 43 kDA
t-tau	Total tau
WM	White matter
WMHs	White matter hyperintensities
