# Supplementary material for: Comorbid Pathologies and Their Impact on Dementia with Lewy Bodies—Current View
Source: Int J Mol Sci. 2025 Aug 8;26(16):7674. doi: 10.3390/ijms26167674 (PMC12386235; doi:10.3390/ijms26167674)
Supplement: Supplementary file 1 [file ijms-26-07674-s001.zip › ijms-3780217-supplementary.pdf]

International Journal of Molecular Sciences (Int. J. Mol. Sci.)
Special Issue "Recent Advances in Dementia with Lewy Bodies and Alzheimer's Disease"

Review

Comorbid Pathologies and Their Impact on Dementia with Lewy Bodies–Current View

Kurt A. Jellinger

Institute of Clinical Neurobiology, Vienna, Austria

Supplementary Appendix: Literature research strategy

Since this is a narrative and not a systematic review, the paper did not follow the guidelines to the items for systematic reviews and meta-analysis protocol (PRISMA-P) (Moher et al. 2015). PubMed, Google Scholar, and Scopus electronic databases were searched for articles in English, published between 2000 and March 2025. Since the review is not concerned with therapies, Cochran Library was not used.

The following criteria were used:

1. Inclusion criteria: Search according to the following key words:

dementia with Lewy bodies, Lewy body dementia, diffuse Lewy body disease, copathologies, comorbidities, Alzheimer disease, Alzheimer-related pathology, cerebrovascular pathology, frontotemporal lobe degeneration, tau-pathologies, neurodegenerative diseases, cerebral microbleeds, cerebral amyloid angiopathy TDP-43 pathology, argyrophilic grain disease, ARTAG, LATE, β-amyloid, tauprotein, α-synuclein, synucleinopathies, hydrocephalus, CSF and plasma biomarkers, MRI biomarkers, Lewy pathology, diabetes mellitus, concomitant findings, white matter lesions, pathophysiology, risk factors, neuroimaging, autoimmune disorders, cardiovascular disease, hypotension.

The results were further screened by title, and only articles containing the above subjects and those in English or with English abstract were included.

2. Exclusion criteria: Conference reports, letters, commentaries, duplicate studies, non-English articles without English abstracts. Studies on diseases other than vascular and mixed parkinsonism or any other studies that did not fit the scope of this review.

The titles and abstracs of the retrieved articles were reviewed by the author to determine the presence of the above mentioned criteria. Subsequently, the selected articles (more than 500) were extracted and reviewed electronically by the author using the abstract and, if available, the full article.

References:

1. Moher, D., Shamseer, L., Clarke, M., Ghersi, D., Liberati, A., Petticrew, M., Shekelle, P., Stewart, L.A, PRISMA-P Group. Preferred reporting items for systematic review and meta-analysis protocols (PRISMA-P) 2015 statement. *Syst Rev* **2015**, *4*, 1. https://doi.org/10.1186/2046-4053-4-1.
